# Cold temperature blocks thyroid hormone-induced changes in lipid and energy metabolism in the liver of *Lithobates catesbeianus* tadpoles

**DOI:** 10.1186/s13578-016-0087-5

**Published:** 2016-03-15

**Authors:** Shunsuke Suzuki, Koichiro Awai, Akinori Ishihara, Kiyoshi Yamauchi

**Affiliations:** Department of Biological Science, Graduate School of Science, Shizuoka University, Ohya 836, Suruga-ku, Shizuoka, 422-8529 Japan; Green Biology Research Division, Research Institute of Green Science and Technology, Shizuoka University, Ohya 836, Suruga-ku, Shizuoka, 422-8529 Japan

**Keywords:** Tadpole, Cold exposure, Thyroid hormone, Glycerophospholipid, Fatty acid, Desaturase, *Lithobates catesbeianus*

## Abstract

**Background:**

Exposure of the American bullfrog *Lithobates catesbeianus* tadpoles to low temperature affects many biological processes including lipid metabolism and the thyroid hormone (TH) signaling pathway, resulting in arrest of TH-induced metamorphosis. To clarify what molecular events occur in this phenomenon, we investigated the glycerophospholipid and fatty acid (FA) compositions, the activities of mitochondrial enzymes and the transcript levels of related genes in the liver of control (26 °C) and cold-treated (4 °C) tadpoles with or without 5 nM 3,3′,5-triiodothyronine (T3).

**Results:**

Exposure to T3 decreased the tail height and polyunsaturation of FAs in the glycerophospholipids, and increased plasma glucose levels and transcript levels of primary TH-response genes including TH receptor, and some energy metabolic (cox4, srebp1 and fas) and FA chain elongase genes (elovl3 and elovl5). However, these T3-induced responses were abolished at 4 °C. Exposure to cold temperature enhanced plasma glucose, triglyceride and free FA levels, monounsaturation of FAs, mitochondrial enzymes activities (cytochrome c oxidase and carnitine palmitoyltransferase; U/g liver), with the upregulation of the genes involved in glycogenolysis (pygl), gluconeogenesis (pck1 and g6pc2), FA β-oxidation (acadl), and cholesterol uptake and synthesis (hmgcr, srebp2 and ldlr1), glycerophospholipids synthesis (pcyt1, pcyt2, pemt, and pparg), and FA monounsaturation (scd1) and chain elongation (elovl1 and elovl2). T3 had little effect on the cold-induced changes.

**Conclusions:**

Our study demonstrated that exposures to T3 and cold temperature exert different effects on lipid metabolism, resulting in changes in the FA composition in glycerophospholipids, and suggests that a cold-induced signal may block TH-signaling pathway around primary TH-response genes.

**Electronic supplementary material:**

The online version of this article (doi:10.1186/s13578-016-0087-5) contains supplementary material, which is available to authorized users.

## Background

Low temperatures profoundly affect physiological responses in animals, especially ectothermic animals, primarily due to the slowing of biochemical reaction rates. Amphibian metamorphosis is highly sensitive to temperature [1], even though it is obligatorily controlled by thyroid hormone (TH). In cold areas, such as higher latitudes or mountain ranges, the American bullfrog, *Lithobates catesbeianus* (formerly known as *Rana catesbeiana*), can grow as tadpoles for 1–3 years before they start to metamorphose [2].

Amphibian metamorphosis can be experimentally arrested at 4 or 5 °C even if tadpoles are treated with the active form of TH, 3,3′,5-triiodothyronine (T3) [1, 3]. Major T3 actions are mediated by nuclear TH receptors (TRs) [4], which up- or down-regulate primary and secondary TH-response genes. Tissue-specific activation or suppression of these TH-response genes may affect gene regulation cascades responsible for tissue-specific transformation during metamorphosis. Findings from recent in vivo and in vitro studies revealed that transcription of the TH-response genes TRβ (a primary TH-response gene) and ornithine transcarbamylase (a secondary TH-response gene) remains at basal levels when *L. catesbeianus* tadpoles and cells are exposed to cold temperature (4 °C) in the presence of T3 for 3‒6 days [3, 5]. However, in zebrafish, TH signal may still be active at low temperature. Findings from a recent report demonstrated that T3 and its metabolite 3,5-diiodothyronine affected swimming performance, metabolic rate, and tissue-specific regulatory enzyme activities, depending on the actual temperature and thermal history of the zebrafish [6]. Whether TH signaling is completely blocked when tadpole metamorphosis is arrested by exposure to cold temperature is not known.

Temperature affects the integrity and fluidity of biological membranes, which are determined by the glycerophospholipid composition of membranes and the fatty acid (FA) composition of the membrane glycerophospholipids [7]. Ectothermic organisms are able to adapt to cold temperature by changes in lipid metabolism. The most well-known and consistent response to cold temperature is an increase in the unsaturation of FAs in glycerophospholipids. Acyl-CoA Δ^9^ desaturase (stearoyl-CoA desaturase), which introduces a double bond in the Δ^9^ position of acyl-CoA, is the enzyme that is responsible for this response to cold temperatures and has been studied in detail in yeast [8] and in fish [9–11]. In addition, significant changes in the gylcerophospholipid composition and the FA composition of glycerophospholipids have been reported in several species of fish with exposure to cold temperature for 2‒7 days [9, 11, 12]. These changes in lipid composition at cold temperatures may optimize the fluidity of the membranes and influence the activity of membrane proteins.

This study was conducted to clarify what effect(s) TH has on the composition of membrane glycerophospholipids and FAs and transcript levels of genes involved with energy and lipid metabolism in *L. catesbeianus* tadpoles, what effect(s) cold temperature (4 °C) has on any changes induced by TH, and what effect(s) cold temperature has independent of TH. In addition, we assessed whether TH counters or enhances the response to cold temperature. Animals were reared in the presence or absence of T3 at 4 or 26 °C (control temperature). The glycerophospholipid composition of hepatic membranes and the FA composition of glycerophospholipids were analyzed by thin-layer chromatography (TLC) followed by gas chromatography (GC), and transcription levels of genes involved with energy and lipid metabolism were quantified by reverse transcription-quantitative polymerase chain reaction (RT-qPCR). As possible effective sites, activities of the enzymes in mitochondrial membrane were also assayed.

## Results

### Morphological and plasma biochemical parameters

Tail height was the most sensitive of the morphological parameters tested to T3 (Additional file 1: Table S1). Exposure to T3 at 26 °C significantly reduced the tail height at day 3, and body weight and length, and tail length and height at day 7. However, the reductions in morphological parameters observed at 26 °C following exposure to T3 were absent at 4 °C. In tadpoles that were not exposed to T3, exposure to cold temperature did not affect these morphological parameters at day 3 or 7 (Additional file 1: Table S1).

**Fig. 1:**
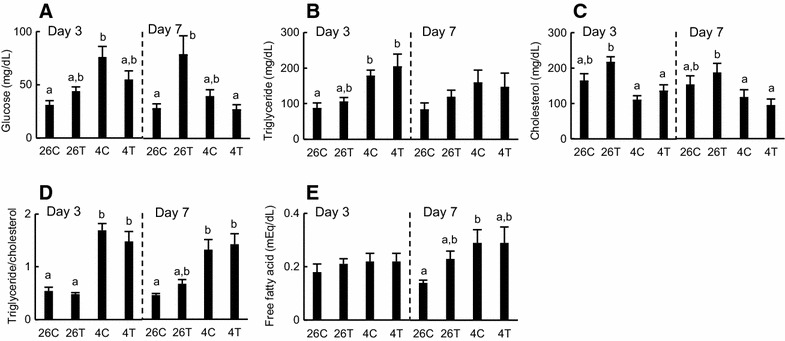
Plasma biochemical parameters in the liver of tadpoles. Tadpoles (*n* = 8/group) were reared in dechlorinated tap water at 26 °C with or without 5 nM 3,3′,5-triiodothyronine (T3; *26C* and *26T*, respectively) or 4 °C with or without 5 nM T3 (*4C* and *4T*, respectively) for 3 days (*left*) or 7 days (*right*). The concentrations of glucose (**A**), triglyceride (**B**), cholesterol (**C**) and free fatty acid (**E**) in plasma were quantified, and the ratio of triglyceride/cholesterol (**D**) was calculated. Values are mean ± SEM (*n* = 8). *Different letters* indicate significantly different means and were determined by one-way analysis of variance with Fisher’s test for multiple comparisons (*p* < 0.05). These experiments were repeated two times, with similar results

Exposure to T3 and/or cold temperature had variable effects on plasma biochemical parameters in tadpoles (Fig. 1). By day 7 at 26 °C, exposure to T3 increased the plasma concentration of glucose (*p* < 0.05). This effect of T3 on the glucose concentration disappeared at 4 °C on day 7. Glucose and triglyceride (TG) concentrations were 2.0-fold to 2.5-fold higher at 4 °C than at 26 °C on day 3 (*p* < 0.05); however, these increases in concentrations were not statistically significant on day 7, and were not affected by T3. On both days 3 and 7, the plasma concentration of cholesterol did not differ significantly among the four groups. Exposure to cold temperature, but not to T3, greatly affected the ratio of TG/cholesterol (3-fold) in plasma. Neither exposure to T3 nor cold temperature (4 °C) altered the concentration of free FAs in plasma, except in the cold-treated tadpoles on day 7.

### Hepatic glycerophospholipid composition and the FA composition of glycerophospholipids

**Fig. 2:**
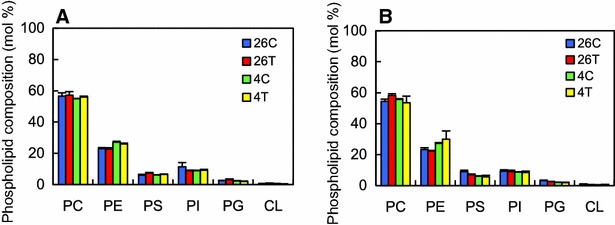
Glycerophospholipid composition in the liver of tadpoles. Tadpoles (*n* = 6/group) were reared in dechlorinated tap water at 26 °C with or without 5 nM 3,3′,5-triiodothyronine (T3; *26C* and *26T*, respectively) or 4 °C with or without 5 nM T3 (*4C* and *4T*, respectively) for 3 days (**A**) or 7 days (**B**). Glycerophospholipids analyzed were phosphatidylcholine (PC), phosphatidylethanolamine (PE), phosphatidylserine (PS), phosphatidylinositol (PI), phosphatidylglycerol (PG), and cardiolipin (CL). Each glycerophospholipid sample was extracted from the livers of two animals. Glycerophospholipid composition is expressed as a percentage of the total moles of glycerophospholipids investigated in each sample. Values are mean ± SEM (*n* = 3)

Of the glycerophospholipids investigated in tadpole liver, more than 50 % were phosphatidylcholine (PC) and approximately 20 % were phosphatidylethanolamine (PE). Both phosphatidylserine (PS), phosphatidylinositol (PI) comprised about 10 % of the glycerophospholipids, whereas phosphatidylglycerol (PG) comprised 3 % and cardiolipin (CL) less than 1 %. The glycerophospholipid composition did not differ significantly among the four groups on both days 3 (Fig. 2A) and 7 (Fig. 2B).

For total glycerophospholipids (Table 1), exposure to T3 decreased the relative abundance of 20:5n-3 at day 7, however, this T3-induced decrease was not observed at 4 °C. Exposure to cold temperature decreased the relative abundance of 20:4n-6 at day 7. T3 did not affect this cold-induced reduction. For each glycerophospholipid (Additional file 2: Tables S2-1 to S2-6), exposures to T3 and cold temperature had only a modest effect on the FA composition at both days 3 and 7, except for PG (Additional file 2: Table S2-5), in which exposure to cold temperature enhanced the proportion of 18:1n-9 by 10-fold at day 3 and 4-fold at day 7. T3 did not affect this cold-induced reduction.

**Table 1:** Changes in compositions of fatty acids in total glycerophospholipids from the tadpole liver

FA	3-day treatment				7-day treatment			
	26 °C		4 °C		26 °C		4 °C	
	T3 (−)	T3 (+)	T3 (−)	T3 (+)	T3 (−)	T3 (+)	T3 (−)	T3 (+)
SFA								
16:0	33.4 ± 0.5	32.9 ± 0.4	31.6 ± 0.2	32.0 ± 0.1	34.1 ± 0.7	33.5 ± 0.3	29.6 ± 0.6	31.6 ± 1.8
18:0	17.9 ± 1.2	17.3 ± 0.6	15.9 ± 0.1	15.4 ± 0.5	16.6 ± 0.4	18.4 ± 1.3	16.8 ± 0.2	18.6 ± 1.7
MUFA								
16:1n-7	5.8 ± 0.4	6.0 ± 0.3	7.8 ± 0.1	7.2 ± 0.1	5.5 ± 0.4	5.9 ± 0.7	5.7 ± 0.1	5.4 ± 0.3
18:1n-9	10.4 ± 0.8^a^	11.6 ± 0.6^a,b^	11.7 ± 0.1^a,b^	13.1 ± 0.2^b^	8.2 ± 0.4	11.0 ± 1.3	10.0 ± 0.6	9.7 ± 0.8
C18 PUFA								
18:2n-6	6.3 ± 0.2	7.5 ± 0.4	7.0 ± 0.3	6.9 ± 0.2	7.2 ± 0.6	6.9 ± 0.4	7.7 ± 0.4	6.8 ± 0.7
18:3n-3	4.6 ± 0.3	5.7 ± 0.3	5.4 ± 0.5	5.3 ± 0.3	6.7 ± 0.9	6.5 ± 1.6	10.7 ± 0.5	9.1 ± 1.0
C20 PUFA								
20:4n-6	9.8 ± 0.8	8.6 ± 0.4	8.2 ± 0.1	7.6 ± 0.3	10.5 ± 0.3^a^	8.7 ± 0.6^a,b^	8.4 ± 0.3^b^	8.1 ± 0.3^b^
20:5n-3	7.0 ± 0.2	5.4 ± 0.1	6.9 ± 0.2	6.5 ± 0.3	5.5 ± 0.3^a^	3.7 ± 0.1^b^	5.8 ± 0.2^a^	5.7 ± 0.7^a^
C22 PUFA								
22:5n-3	2.1 ± 0.1	2.3 ± 0.1	2.5 ± 0.2	2.5 ± 0.1	2.4 ± 0.2	2.6 ± 0.1	2.5 ± 0.1	2.4 ± 0.1
22:6n-3	2.9 ± 0.3	2.8 ± 0.1	3.0 ± 0.2	3.7 ± 0.1	3.2 ± 0.3	2.8 ± 0.3	2.8 ± 0.1	2.6 ± 0.2

18:1n-9 contains18:1n-9t (elaidic acid) and 18:1n-9c (oleic acid). Each value is the mean ± SEM (n = 3). *Different letters* indicate significantly different means and were determined by one-way analysis of variance with Fisher’s test for multiple comparisons (*p* < 0.05)

*FA* fatty acid, *T3* 3,3′,5-triiodothyronie, *SFA* saturated fatty acid, *MUFA* monounsaturated fatty acid, *PUSA* polyunsaturated fatty acid

**Fig. 3:**
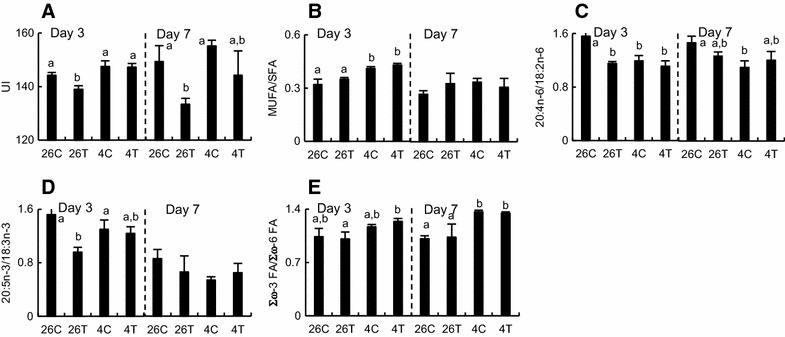
Unsaturation and utilization of the fatty acids of glycerophospholipids in the liver of tadpoles. Tadpoles (*n* = 6/group) were reared in dechlorinated tap water at 26 °C with or without 5 nM 3,3′,5-triiodothyronine (T3; *26C* and *26T*, respectively) or 4 °C with or without 5 nM T3 (*4C* and *4T*, respectively) for 3 days (*left*) or 7 days (*right*). Each glycerophospholipid sample was extracted from the livers of two animals. The unsaturation index (UI, **A**), the ratios of monounsatureated fatty acid (MUFA)/saturated fatty acid (SFA) (**B**), 20:4n-6/18:2n-6 (**C**), 20:5n-3/18:3n-3 (**D**) and Σω-3 FA/Σω-6 FA (**E**) were calculated from the fatty acid composition of glycerophospholipids (Table 1). Values are mean ± SEM (*n* = 3). *Different letters* indicate significantly different means and were determined by one-way analysis of variance with Fisher’s test for multiple comparisons (*p* < 0.05)

Exposure to T3 decreased the unsaturation index (UI, the average number of double bonds per 100 FA molecules) in total glycerophospholipids on days 3 and 7 (Fig. 3A). The T3-induced decrease was not detected at 4 °C. The UI value of FAs did not significantly differ between the control and cold-treated tadpoles in the absence of T3.

The ratio of monounsaturated FA (MUFA)/saturated FA (SFA), which partially reflects Δ^9^ desaturase activity, was not changed in total glycerophospholipids with exposure to T3 (Fig. 3B), whereas it increased significantly on day 3 with exposure to cold temperature. The cold-induced response was not observed on day 7, and was not affected by T3.

The ratio of 20:4n-6/18:2n-6 and/or the ratio of 20:5n-3/18:3n-3, both of which partially reflect the sequential reactions consisting of the Δ^6^ desaturase and very long chain FA elongase activity of the ω-6 and ω-3 polyunsaturated FAs (PUFAs), respectively (see Additional file 3: Figure S1B), decreased in total glycerophospholipids with exposures to T3 and cold temperature on day 3 or 7 (Fig. 3C, D). These T3-induced or cold-induced responses were not enhanced additively or synergistically at 4 °C and with T3.

The ratio of Σω-3 FA/Σω-6 FA, which indicates a preference of the ω-3 and ω-6 FA utilization in glycerophospholipids, increased in total glycerophospholipids (Fig. 3E) at day 7 with exposure to cold temperature but not with exposure to T3. Exposure to T3 did not affect this cold-induced response.

### Hepatic mitochondrial membrane enzymes activity

**Fig. 4:**
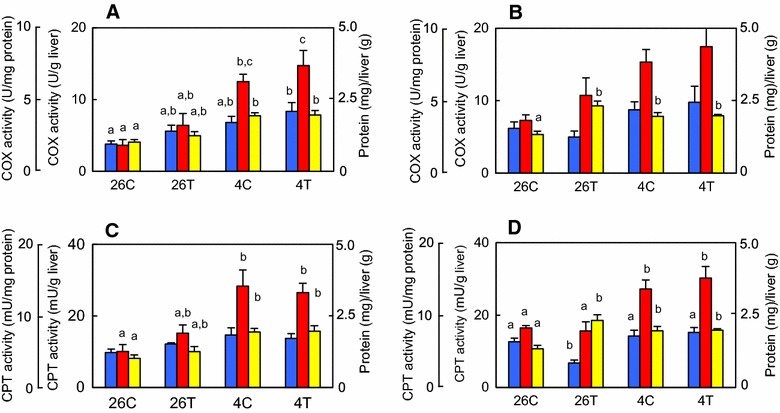
Mitochondrial enzyme activity in the liver of tadpoles. Tadpoles (*n* = 8/group) were reared in dechlorinated tap water at 26 °C with or without 5 nM 3,3′,5-triiodothyronine (T3; *26C* and *26T*, respectively) or at 4 °C with or without 5 nM T3 (*4C* and *4T*, respectively) for 3 days (**A**, **C**) or 7 days (**B**, **D**). **A**, **B** Cytochrome c oxidase (COX) activity and the membrane protein amount. **C**, **D** Carnitine palmitoyltransferase (CPT) activity and membrane protein amount. Each assay was carried out using mitochondria-rich fractions obtained from the livers of two animals. Values are mean ± SEM (*n* = 4). The activities are presented as either units per mg of membrane protein (U or mU/mg protein, *blue*) or units per g of liver (U or mU/g liver, *red*). Membrane protein amounts are presented as mg per g of liver [protein (mg)/liver (g), *yellow*]. *Different letters* indicate significantly different means among the four groups and were determined by one-way analysis of variance with Fisher’s test for multiple comparisons (*p* < 0.05). Significant difference between the enzyme activities (U or mU/g liver) and membrane protein amounts were detected on day 3 (**A**, *p* < 0.01; **C**, *p* < 0.05) but not on day 7 (**B** and **D**) by two-way analysis of variance with Fisher’s test for multiple comparisons. These experiments were repeated two times, with similar results

Cytochrome c oxidase (COX) and carnitine palmitoyltransferase (CPT) activities, when estimated as U/mg membrane protein, did not differ significantly among the four treatment groups (Fig. 4). However, when estimated as U/g wet weight of the liver used, although COX and CPT activities did not significantly increase with exposure to T3, these activities increased 2.8-fold to 3.4-fold with exposure to cold temperature at day 3 (Fig. 4A, B), indicating that the amount of mitochondrial protein per liver wet weight was increased by exposure to cold temperature. This increase in enzyme activities was significantly greater than that in the ratio of membrane protein (mg) to liver (g), 1.9, at least on day 3. Exposure to T3 did not affect the cold-induced response.

### Transcript amounts of hepatic genes

#### Primary TH-response gene transcripts

**Fig. 5:**
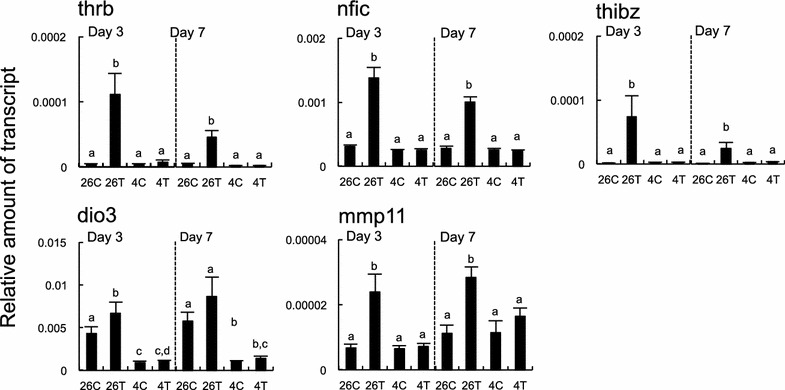
Effects of 3,3′,5-triiodothyronine and cold exposures on primary thyroid hormone-response gene transcripts. Reverse transcription-quantitative polymerase chain reaction analyses were conducted with RNAs from the liver of the tadpoles (*n* = 6/group) that were reared with or without 5 nM 3,3′,5-triiodothyronine (T3) at 26 °C (26C and 26T) or 4 °C (4C and 4T) for 3 days (*left*) or 7 days (*right*). The genes tested are: thyroid hormone receptor TRβ (thrb), nuclear factor I/C (nfic), thyroid hormone induced bZip protein (thibz), deiodinase 3 (dio3), and matrix metallopeptidase 11 or stromelysin-3 (mmp11). The data are mean ± SEM (*n* = 6). *Different letters* indicate significantly different means (*p* < 0.05). These experiments were repeated two times, with similar results

Five TH-response genes that are known to respond directly to TH through TH response elements in *X. laevis* or *L*. *catesbeianus* (thrb, nfic, thibz, dio3 and mmp11) [3, 5, 13], except for the dio3 on day 7, were upregulated with exposure to T3. However, the T3-induced responses were not observed at 4 °C (Fig. 5).

#### Energy metabolism gene transcripts

**Fig. 6:**
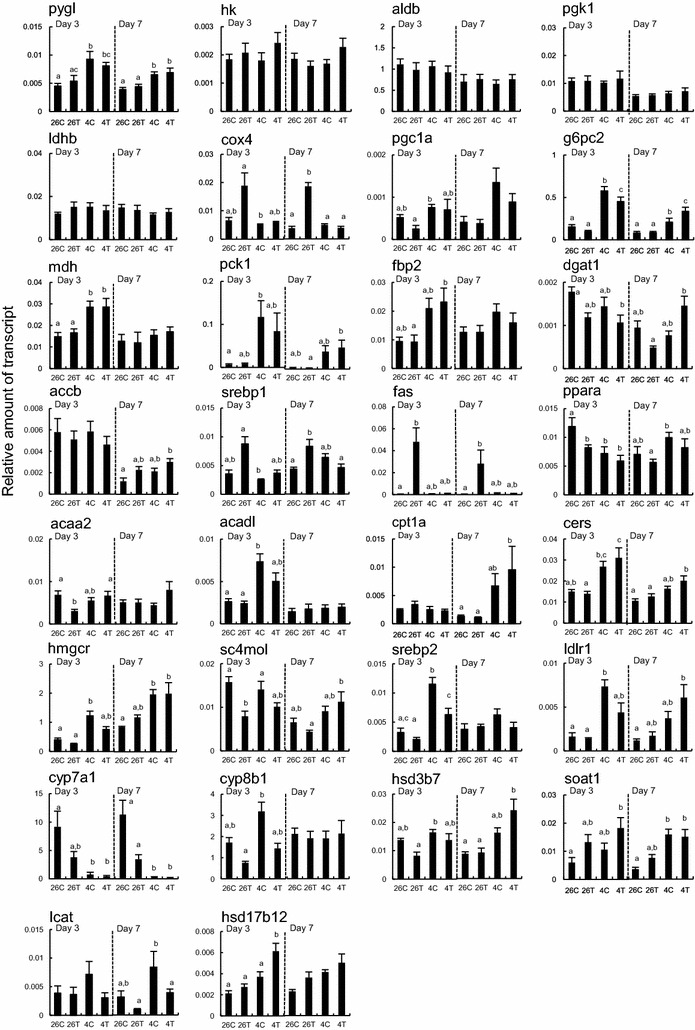
Effects of 3,3′,5-triiodothyronine and cold exposures on energy metabolic gene transcripts. Reverse transcription-quantitative polymerase chain reaction analyses were carried out with RNAs from the liver of the tadpoles (*n* = 6/group), as described in Fig. 5. The genes tested are: phosphorylase, glycogen, liver (pygl), hexokinase (hk), aldolase B (aldb), phosphoglycerate kinase 1 (pgk1), lactate dehydrogenase B (ldhb), cytochrome c oxidase subunit IV (cox4), glucose-6-phosphatase, catalytic, 2 (g6pc2), malate dehydrogenase (mdh), phosphoenolpyruvate carboxykinase 1 (pck1) and fructose-1,6-bisphosphatase 2 (fbp2), belonging to the pathways of the glycolysis, TCA, electron transport system, and glyconeogenesis; diacylglycerol *O*-acyltransferase 1 (dgat1), acetyl-CoA carboxylase β (accb), FA synthase (fas), acetyl-CoA acyltransferase 2 (acaa2), long chain-acyl-CoA dehydrogenase, (acadl), carnitine palmitoyltransferase 1 (cpt1a) and ceramide synthase (cers), belonging to the pathways of the biosynthesis and metabolism of FAs, TGs and ceramides; 3-hydroxy-3-methyl-glutaryl-CoA reductase (hmgcr), sterol-C4-methyl oxidase–like (sc4mol), low density lipoprotein receptor 1 (ldlr1), cytochrome P450 7A1 (cyp7a1), cytochrome P450 8B1 (cyp8b1), 3β-hydroxy-Δ5-C27-steroid dehydrogenase type 7 (hsd3b7), sterol *O*-acyltransferase 1 (soat1), lecithin-cholesterol acyltransferase (lcat), hydroxysteroid (17-β) dehydrogenase 12 (hsd17b12), belonging to the pathways of the biosynthesis, metabolism and transport of cholesterols, steroids and bile acids; and the transcription regulatory proteins, which are peroxisome proliferator-activated receptor γ coactivator 1α (pgc1a), sterol regulatory element-binding transcription factor 1 (serbp1), sterol regulatory element-binding transcription factor 2 (serbp2) and peroxisome proliferator-activated receptor α (ppara). The data are mean ± SEM (*n* = 6). *Different letters* indicate significantly different means (*p* < 0.05). These experiments were repeated two times, with similar results

The effect of exposure to T3 or cold temperature was variable on transcript amounts of energy metabolism genes. We detected 6 TH-response genes (cox4, srebp1, fas, ppara, acaa2 and sc4mol) and 11 cold-response genes (pygl, g6pc2, mdh, pck1, ppara, acadl, hmgcr, srebp2, ldlr1, cyp7a1 and soat1) at day 3 or 7 in the tadpole liver (Additional file 4: Figure S2). The T3-induced responses were not observed at 4 °C, whereas most of the cold-induced responses were not affected by exposure to T3 (Fig. 6).

There was no change in the transcript amount of the glycogen phosphorylase gene (pygl) with exposure to T3, however, it increased with exposure to cold on days 3 and 7, suggesting the enhancement of glycogenolysis in the liver by cold stimuli. No effects of cold and T3 exposures on days 3 and 7 were detected on the transcript amounts of four genes in the glycolysis pathway (hk, aldb, pgk1 and ldhb). Exposure to T3 at 26 °C but not at 4 °C increased the amounts of the cox4 transcript on days 3 and 7 without significant effects on the pgc1a expression, whereas cold exposure increased the transcript amounts of three genes involved in gluconeogenesis (g6pc2, mdh and pck1) on at least day 3 regardless of whether tadpoles were exposed to T3 or not.

Neither exposure to T3 nor cold temperature affected the transcript amount of the gene that is involved in TG synthesis (dgat1). Of the three genes that are involved in FA synthesis (accb, srebp1 and fas), the srebp1 and fas transcript levels were high on at least day 7 of exposure to T3 at 26 °C but not at 4 °C, although the accb transcript levels were affected by neither exposure to T3 nor cold temperature.

There was a reduction in the transcript amounts of the transcription factor ppara gene, which plays an important role in β-oxidation, and the acaa2 gene at 26 °C on day 3 with exposure to T3. However, there was no change in the transcript amounts of the other β-oxidation genes (acadl and cpt1a) and ceramide synthase gene (cers). Exposure of cold temperature reduced significantly the transcript amount of ppara and enhanced the transcript amount of acadl on day 3. The cold-induced responses were not affected by exposure to T3.

Of the genes involved in cholesterol synthesis and uptake (hmgcr, sc4mol, srebp2 and ldlr1), the amount of the sc4mol transcript decreased with exposure to T3 on day 3 at 26 °C, but this T3 effect was not detected at 4 °C, whereas the amounts of the hmgcr, srebp2 and ldlr1 gene transcripts increased with exposure to cold temperature on at least day 3. Exposure to T3 did not affected most of the cold-induced increases.

At 26 °C, exposure to T3 did not have a significant effect on the transcript levels of three genes involved in bile synthesis (cyp7a1, cyp8b1 and hsd3b7), two genes involved in the synthesis of cholesterol ester to accumulate it as cytoplasmic lipid droplets (soat1) or to transport it via blood lipoproteins into the liver (lcat), or one gene involved in estradiol and arachidonic acid synthesis (hsd17b12) [14]. The cyp7a1 transcript amount decreased on days 3 and 7 with exposure to cold temperature. In contrast, the cyp8b1, hsd3b7, lcat and hsd17b12 transcript amounts were hardly affected by cold exposures on days 3 and 7. The soat1 transcript amount increased on day 7 of cold exposure. These cold-induced changes were not affected by exposure to T3.

#### Glycerophospholipid metabolism gene transcripts

**Fig. 7:**
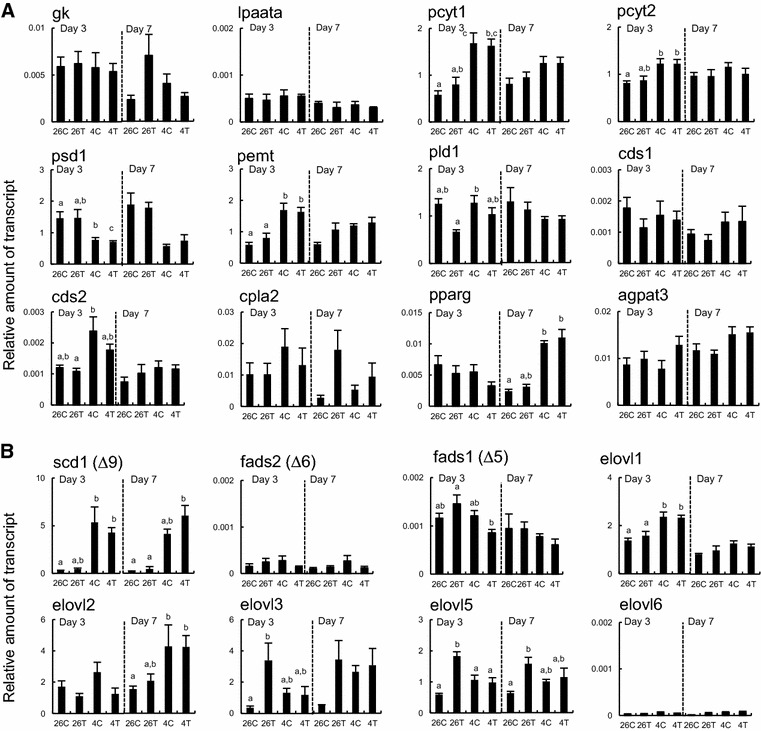
Effects of 3,3′,5-triiodothyronine and cold exposures on gene transcripts for glycerophospholipid synthesis (**A**) and very long chain fatty acid desaturation and elongation (**B**). Reverse transcription-quantitative polymerase chain reaction analyses were carried out with RNAs from the liver of the tadpoles (*n* = 6/group), as described in Fig. 5. The genes tested are: glycerol kinase (gk), lysophosphatidic acid acyltransferase-α (lpaata), phosphate cytidylyltransferase 1 (pcyt1), phosphate cytidylyltransferase 2 (pcyt2), phosphatidylserine decarboxylase 1 (psd1), phosphatidylethanolamine N-methyltransferase (pemt), phospholipase D1 (pld1), phosphatidate cytidylyltransferase 1 (cds1), phosphatidate cytidylyltransferase 2 (cds2), belonging to the de novo synthesis pathway of glycerophospholipids; cytosolic phospholipase A2 IVB (cpla2), the transcription factor (pparg) and 1-acylglycerol-3-phosphate *O*-acyltransferase 3 (agpat3), belonging to the remodeling pathway (cycle of deacylatiom/reacylation) in **A**; stearoyl-CoA desaturase or Δ^9^ desaturase (scd1), Δ^6^ desaturase or fatty acid (FA) desaturase 2 (fads2), Δ^5^ desaturase or FA desaturase 1 (fads1), and the enzymes for elongation of very long chain FAs (elovl1, elovl2, elovl3, elovl5 and elovl6) in **B**. The data are mean ± SEM (n = 6). *Different letters* indicate significantly different means (*p* < 0.05). These experiments were repeated two times, with similar results

The effect of exposure to T3 was not observed, however the effect of exposure to cold temperature was variable on transcript amounts of glycerophospholipid metabolism genes (Additional file 3: Figure S1A). We detected four cold-induced or -suppressed genes (pcyt1, pcyt2, psd1 and pemt) at day 3 and one cold-induced gene (pparg) at day 7. Exposure to T3 did not alter these cold-induced responses (Fig. 7A).

In the de novo glycerophospholipids synthesis pathway, the pcyt1, pcyt2 and pemt transcript amounts were significantly up-regulated whereas the psd1 transcript amount was down-regulated on day 3 of cold exposure. However, these effects were lost on day 7. Cold exposure on day 7 enhanced the amount of the pparg transcript that encodes a receptor for 20:4n-6 metabolites. There were no effects of exposure to cold temperature on the expression of the other genes tested: those involved in phosphatidic acid synthesis (gk and lpaata), the de novo gylcerophospholipid synthesis (pld1, cds1 and cds2), the remodeling pathway (reacylation of membrane glycerophospholipids from glycerolysophospholipids) (cpla2 and agpat3).

#### Very long chain FA desaturation and elongation gene transcripts

The effect of exposure to T3 or cold temperature was variable on transcript amounts of very long chain FA desaturase and elongase genes (Additional file 3: Figure S1B), in which 2 TH-response genes (elovl3 and elovl5) and 3 cold-response genes (scd1, elovl1 and elovl2) were detected in the tadpole liver. The T3-induced responses at 26 °C were not observed at 4 °C whereas the cold-induced responses were not affected by exposure to T3 (Fig. 7B).

Among the three desaturase genes tested, the effect of exposure to T3 was not detected in these transcript amounts. The amounts of Δ^9^ desaturase (scd1) transcript, but not the Δ^6^ (fads2) and Δ^5^ (fads1) desaturase transcripts, increased on at least day 3 of cold exposure. Among the five elovl genes tested, the elovl3 and elovl5 transcript amounts increased on at least day 3 with exposure to T3 at 26 °C, whereas the elovl1 and elovl2 transcript amounts increased on day 3 and day 7, respectively, with exposure to cold temperature. Neither exposure to T3 nor cold temperature affected significantly the amount of the elovl6 transcript.

## Discussion

The present study demonstrates that exposures to T3 and cold temperature for up to 7 days have different effects on components of membrane lipids with dynamic changes in transcript levels for energy/carbohydrate/lipid metabolism (Table 2). Exposure of tadpoles to T3 at 26 °C induced primary and secondary TH-response genes, resulting in tail regression. In the liver, remodeling of membrane lipids, FA synthesis and mitochondrial activity were activated with elevated plasma glucose level, while lipolysis and sterol synthesis were suppressed. Exposure to cold temperature and exposures to both cold temperature and T3 induced similar changes in the liver: remodeling of membrane lipids, lipogenesis (sterol and/or ceramide synthesis), lipolysis, mitochondrial activity, glycogenolysis, probably glyceroneogenesis/TG/FA cycle, with transient elevation of plasma glucose and TG. We detected 13 TH-response genes including at least 5 primary TH-response genes and 19 cold-response genes. Under our experimental conditions, morphological, biochemical and transcriptional changes induced by T3 in the tadpoles reared at 26 °C were not observed at 4 °C, although the majority of the changes induced by cold temperature were not affected by exposure to T3.

**Table 2:** Effects of thyroid hormone and cold exposures on biochemical and transcriptional changes in the liver of *L. catesbeianus* tadpoles

Treatment	Possible events	Biochemical and transcriptional changes
T3(+) at 26 °C		
(Enhancement)	Tail regression	Tail height↓, body weight (7d)↓, body length (7d)↓, tail length (7d)↓
	T3-response genes	thrb↑, nfic↑, thibz↑, dio3 (3d)↑, mmp11↑
	Remodeling of membrane lipids	Unsaturated ω-3 and ω-6 FAs (3d)↓, UI↓, 20:5n-3 (7d) ↓, elovl3 (3d)↑, elovl5↑
	Lipogenesis (FA synthesis)	srebp1 (7d)↑, fas↑
	Glycogenolysis?	Plasma glucose (7d)↑
	Mitochondrial activity	Mitochondrial protein (7d)↑, cox4 (7d)↑
(Suppression)	Lipolysis (β-oxidation)	ppara (3d)↓, acaa2 (3d)↓
	Lipogenesis (sterol synthesis)	sc4mol (3d) ↓
T3(−) at 4 °C		
(Enhancement)	Remodeling of membrane lipids	Unsaturated ω-6 FAs↓, MUFA/SFA (3d)↑, 20:4n-6 (7d) ↓, ω-3 FAs/ω-6 FAs (7d)↑, scd1 (3d)↑, elovl1 (3d)↑, elovl2 (7d)↑, pcyt1 (3d)↑, pcyt2 (3d)↑, pemt (3d)↑, psd1 (3d)↓, pparg (7d)↑
	Sterol synthesis	srebp2 (3d)↑, ldlr1 (3d)↑, hmgcr↑, cyp7a1↓, soat1 (7d)↑
	Lipolysis (β-oxidation)	ppara (3d)↓, acadl (3d)↑, CTP↑
	Gluconeogenesis and glyceroneogenesis/TG/FA cycle	Plasma glucose (3d)↑, TG (3d)↑, TG/Chol↑, FFA (7d)↑, pygl↑, g6pc2↑, pck1 (3d)↑, mdh (3d)↑
	Mitochondrial activity	18:1n-9 in PG↑, mitochondrial protein↑, COX (3d)↑, CPT↑
T3(+) at 4 °C		
(Enhancement)	Remodeling of membrane lipids	Unsaturated ω-6 FAs (3d)↓, MUFA/SFA (3d)↑, 18:1n-9 (3d)↑, 20:4n-6 (7d) ↓, ω-3 FAs/ω-6 FAs (7d)↑, scd1↑, elovl1 (3d)↑, elovl2 (7d)↑, pcyt1 (3d)↑, pcyt2 (3d)↑, pemt (3d)↑, psd1 (3d)↓, pparg (7d)↑
	Sterol and ceramide synthesis	hsd17b12 (3d)↑, cers↑, soat1↑, cyp7a1↓, hmgcr (7d)↑, hsd3b7 (7d)↑, ldlr1 (7d)↑, pparg (7d)↑
	Lipolysis (β-oxidation)	ppara (3d)↓, cpt1a (7d)↑, CPT↑
	Gluconeogenesis and glyceroneogenesis/TG/FA cycle	TG (3d)↑, TG/Chol↑, pygl↑, g6pc2↑, mdh (3d)↑
	Mitochondrial activity	18:1n-9 in PG↑, mitochondrial protein↑, COX (3d)↑, CPT↑
(Unclear)	FA/TG synthesis	dgat1 (3d)↓, accb (7d)↑

No parenthesis of the column “Biochemical and transcriptional changes” indicates significant changes on both day 3 and day 7. Gene names are shown in Additional file 5: Table S3.

*Chol* cholesterol, *COX* cytochrome c oxidase, *CPT* carnitine palmitoyltransferase, *FA* fatty acid, *FFA* free fatty acid, *MUFA* monounsaturated fatty acid, *PG* phosphatidylglycerol, *SFA* saturated fatty acid, *TG* triglyceride, *UI* unsaturation index. Parentheses indicate the day showing signifcant changes: *3d* on day 3, *7d* on day 7

### Effects of exposures to T3 and cold temperature on the FA composition of glycerophospholipids

In contrast to the glycerophospholipid composition which was hardly affected by exposure to T3 or cold temperature, the FA compositions of the total glycerophospholipids and specific classes of the glycerophospholipids were variously changed in response to these stimuli. At least three different steps may contribute to these changes: (1) the monounsaturation of FAs, which is deduced from the ratio of MUFA/SFA, predominantly depending on the Δ^9^ desaturase activity, (2) the polyunsaturation of FAs, which are deduced from the ratios of 20:4n-6/18:2n-6 and 20:5n-3/18:3n-3, probably reflecting sequential reactions consisting of Δ^6^ desaturase, very long chain FA elongase ELOVL5 and Δ^5^ desaturase (Additional file 3: Figure S1B), and (3) the acylation and deacylation that determine the preference for FA classes in the glycerophospholipids, which the ratio of Σω-3 FA/Σω-6 FA presumably reflected, in the de novo synthesis pathway and the cycle of acylation and deacylation (Additional file 3: Figure S1A). Exposure to T3 suppressed the step (2) alone, leading to the decline in the UI value; whereas exposure to cold temperature enhanced the steps (1) and (3) but suppressed the step (2), leading to no change in the UI value.

Changes in the UI values and FA ratios described above are also reported in T3-treated mice [15] and cold-tolerant fishes [12, 15]. However, there are marked differences in these responses among the species. The UI value and the MUFA/SFA, 20:4n-6/18:2n-6, 20:5n-3/18:3n-3, Σω-3 FA/Σω-6 FA ratios were elevated in mouse liver in response to T3 [15], unlike tadpole liver. After cold-exposure, the MUFA/SFA ratios increased in carp liver [16] but not in rainbow trout liver [12, 17]. Unlike in the tadpole liver, in carp and rainbow trout livers [12, 16, 17] the 20:4 n-6/18:2n-6 or 20:5 n-3/18:3n-3 ratio increased. The ratio of Σω-3 FA/Σω-6 FA dramatically decreased in carp but increased in rainbow trout liver [12, 16]. These observations suggest that the pathway of very long chain FA unsaturation, elongation or acylation/deacylation may be differently regulated in a species-specific manner to maintain lipid homeostasis or membrane fluidity in response to T3 and cold stimuli. The variety of the species-specific responses may result in the different changes in FA compositions among species.

In this study, we detected glycerophospholipid-specific changes in the FA compositions, i.e., 18:1n-9 in PG, in the cold-treated tadpoles (Additional file 2: Table S2-5). Although it is unclear at present why the elevation in the proportion of 18:1n-9 is notable in PG, a higher turnover rate of PG is likely to provide a possible explanation. Alternatively, glycerophospholipid class-specific acylation and deacylation pathway may contribute to the PG-specific elevation of C18:1n-9 proportion [18]. As PG is a precursor of CL that is the constituent lipid of the mitochondrial inner membrane, the increase in 18:1n-9 composition of PG is likely to affect mitochondrial functions with exposure to cold temperature.

### Effects of exposures to T3 and cold temperature on mitochondrial enzyme activities

Although THs have important roles in mitochondrial function and biogenesis through direct actions of TRs in mitochondria and indirect actions of TRs by which regulatory genes such as pgc1a/b are activated in nucleus and the translational products act as transcription factors in mitochondria in mammals [19], the COX and CPT activities and the pgc1a transcript levels hardly responded to exposure to T3 in the tadpole liver, despite the strong upregulation of the cox4 transcript by T3. Such a discordance between the enzyme activity and its transcript abundance was also reported for fish COX [6, 20], suggesting the presence of non-transcriptional regulation.

Exposure to cold temperature may alter mitochondrial functions probably by increasing the amount of mitochondrial proteins and another unknown mechanism under our experimental conditions. The COX and CPT activities (U/g tissue but not U/mg protein) increased in the liver mitochondria membranes of cold-treated tadpoles, in agreement with a previous report in rainbow trout muscle mitochondria membranes [21]. This increase occurred without the activation of the pgc1a gene, which encodes a master regulator of mitochondrial biogenesis and functions. Therefore, tadpole liver may increase the mitochondrial protein content rather than the copy number of the mitochondrial genome in response to cold stimulus, as proposed previously [20]. Our data also suggest that the cold-induced increase in COX and CPT activities may also be explained by an unknown additional mechanism. It is unclear whether the cold-induced increase in COX and CPT activities is because of changes in the glycerophospholipid milieu in mitochondrial membranes as shown previously in the carp red muscle mitochondria [22] or increases in the amounts of COX and CPT per mitochondrial membrane proteins.

### Effects of exposures to T3 and cold temperature on transcript amounts of TH-response genes

T3-induced transcriptional activation or suppression at 26 °C, including that in 5 primary TH-response genes, was lost in the liver with exposure to cold temperature. In contrast, almost all of cold-induced gene activation or suppression was not affected by exposure to T3. As the primary TH-response genes may have TH response elements in their regulatory regions [13], cold stimuli may affect the T3-dependent activation of these genes through a common molecular mechanism. Our previous studies indicated that exposure to cold temperature abolished the T3-induced increase in the amount of acetylated histone H3 at lysine 9 in the thrb gene [5], suggesting that cold-induced signal may affect a transcriptional step between TR transactivation and the histone modification in at least the thrb gene. It is unclear whether the signal derived from cold stimuli interferes with the transactivation of the liganded TRs or with the interaction of TRs with other transcriptional regulatory proteins such as co-activators, nucleosome remodellers and histone modifiers on TH response elements in the primary TH-response genes. It was recently reported that T3 signaling persists even at 5 °C in at least some primary TH-response genes of the bullfrog tadpoles. These phenomena were tissue-specific and clearly detected in tadpole tail fin [23]. In fish, hypothyroidism impaired swimming performance and metabolic rates in cold-acclimated but not warm-acclimated fish whereas T3- or diiodothyronine-treatment restored these performances [6], indicating that cold acclimation sensitizes TH actions. In the transcriptome analysis of zebrafish larvae exposed to cold temperature [10], rev-erb subfamily of nuclear receptors was proposed as candidate master genes of cold response. However, the bullfrog rev-erb gene transcript was not respond to exposure to cold temperature (data not shown). Further studies about the T3 effects on T3-response genes in the liver of cold-acclimated hypothyroid tadpoles are required.

### Effects of exposures to T3 and cold temperature on energy metabolic pathways

Exposures to T3 and cold temperature dramatically and differently altered the energy metabolisms in the tadpole liver. Exposure to T3 at 26 °C increased plasma glucose level, which may be induced by enhanced glycogenolysis and activation of glucose 6-phosphatase that occur during spontaneous or TH-induced metamorphosis [24–26]. However, our transcriptional data (pygl, g6pdh, mdh and pkc1) did not support T3-dependent glycogenolysis and gluconeogenesis. It is likely that mechanisms other than transcriptional regulation, e.g., the modulation of enzyme activity by phosphorylation, may be involved in the activity of rate-limiting enzymes for glycogenolysis such as glycogen phosphorylase if plasma glucose is mainly derived from the liver. There has been no solid evidence that elevated plasma glucose is derived from the larval tissues such as tail and intestine that are hydrolyzed during metamorphosis. On the other hand, exposure of tadpoles to cold temperature increased plasma glucose level with elevated amounts of the transcripts for pygl, g6pc2, mdh and pck1 on day 3 but not on day 7, suggesting a transient activation of glycogenolysis and gluconeogenesis. This hyperglycemia may have an advantage to acclimate to cold temperature. The bullfrog *L. catesbeianus* [27], as well as other northern frogs: *Rana sylvatica* [28] and *Pseudacris triseriata* [29], resists to freezing for survival at sub-zero temperature, by increasing plasma glucose level as a cryoprotectant. *R. sylvatica* and *P. triseriata* mobilize glucose from liver glycogen.

Increases in plasma TG (on day 3) and free FA (on day 7) levels and in transcript abundance of the hepatic pck1 (on day 7) with exposure to cold temperature suggest that TG/FA cycle may be activated between the liver and the other tissues such as fat bodies. In mammals, this cycle is usually activated under starvation with activation of phosphoenolpyruvate carboxylase in the liver and with its suppression in the adipose tissue [30]. A possible scenario in the tadpoles is that lipids are hydrolysed into free FAs and glycerol in the fat bodies or other tissues [31–34] and then free FAs are re-esterified back to TG either in the fat bodies or in the liver, where glyceroneogenesis is activated to make glycerol 3-phosphate for TG synthesis. Increases in hepatic phosphoenolpyruvate carboxylase activity and plasma glycerol level were reported in the rainbow smelt in response to cold temperature [31]. However, it is unclear why there is a difference in timing of the increases in plasma free FA and TG levels in the tadpoles reared at 4 °C.

Exposure to cold temperature enhanced the transcript abundances of genes for cholesterol, cholesterol esters and/or ceramide syntheses, and suppressed those for cholesterol catabolism into bile acids, with a trend toward a decrease in plasma cholesterol level. This strongly suggests that free sterol level is low in the liver cells. The liver have to provide lipid and cholesterol building blocks to every tissue for extensive reconstruction of membranes. Remodeling of the membrane lipids may be a prerequisite for coping with cold-induced changes in the fluidity of lipid membranes and activity or conformation of membrane proteins [35]. Changes in cholesterol content of lipids may be most critical for short-term cold acclimation [36, 37].

Among the transcripts for transcription regulatory proteins, striking changes were found in the amounts of the srebp1 and srebp2 transcripts, which encode important transcription factors involved in lipogenesis and cholesterol homeostasis, respectively [38]. The srebp1 gene was up-regulated by exposure to T3 whereas the srebp2 gene was up-regulated by exposure to cold temperature. The increase in the ratio of TG/cholesterol in plasma detected with exposure to cold temperature may reflect the increase in cellular uptake of cholesterol from plasma via the ldlr activation by the srebp2. The ppara gene was suppressed or not affected by exposures to T3 and cold temperature with relatively high TG levels in plasma, suggesting that the liver may not be under conditions of energy deprivation [39]. Nevertheless, FA β-oxidation, which is usually activated by the transcription factor PPARα that the ppara gene encodes, was activated with exposure to cold temperature. The underlying molecular mechanism is unclear.

Approximately half of the biochemical and transcriptional changes in response to exposure to cold temperature, are detected on day 3 only (Table 2), and returned to normal on day 7. These may be the transient stress responses to cold stimuli, as seen in mammalian and fish species [40, 41].

### Effects of exposures to T3 and cold temperature on membrane lipid metabolic pathways

Very long chain FAs with >C22 are produced from long chain FAs with >C18, are essential for components of membrane lipids (e.g., sphingolipids and glycerophospholipids) and precursors of lipid mediators. Fatty acyl desaturases and ELOVLs are rate-limiting enzymes for FA chain desaturation and elongation with characteristic substrate-specificity (Additional file 3: Figure S1B). Dysfunction of these mammalian enzymes exert abnormality of lipid synthesis, storage, metabolism in liver or adipose tissues, immune cell homeostasis, inflammatory reactions, and sterility, in an enzyme-specific manner [42]. Although we cannot clearly state the functional meaning of the changes in the transcript amounts of elongases and desaturase genes, the nature of lipid membranes may be specifically altered to optimize the membrane fluidity, structure or activity of membrane proteins, in response to exposure to T3 or cold temperature.

In spite of various changes in the transcript amounts of the metabolic enzymes for glycerophospholipids and very long chain FAs in response to exposures to T3 and cold temperature, there were weak correlations between the transcript amounts and the glycerophospholipid or FA amounts. Only the upregulation of the scd1 gene by cold exposure was correlated with the increase in the MUFA/SFA ratio. In mammals, SREBP1c plays a pivotal role in the activation of the scd1 gene [43]. As the srebp1 gene was not up-regulated by exposure to cold temperature in the tadpole liver, post-translational regulation as found in mammals [42] may be important to activate SREBP1 protein as a transcription factor. The ratios of 20:4n-6/18:2n-6 and 20:5n-3/18:3n-3 declined with exposures to T3 and/or cold temperature, nevertheless the transcript amounts of the genes fads1 and fads2 for Δ^5^ and Δ^6^ desaturases, respectively, which are rate-limiting enzymes for PUFA conversion (Additional file 3: Figure S1B), were not affected by these exposures. In the present study, we have mainly investigated the de novo glycerophospholipid synthesis pathway (Kennedy pathway) (Additional file 3: Figure S1A). However, the remodeling pathway by cycle of deacylation-reacylation of membrane glycerophospholipids (Lands’ cycle) (Additional file 3: Figure S1A), in which a number of enzymes are involved [44], may also profoundly affect the compositions of the FAs in the glycerophospholipids [18].

## Conclusions

We demonstrated that pre-metamorphic tadpoles induced a variety of metabolic changes by short-term exposures to T3 and cold temperature, which include changes in biochemical parameters in plasma, and FA compositions of the glycerophospholipids, mitochondrial activities and transcript levels of the genes for energy and lipid metabolisms in the liver. In particular, the pathway of the FA desaturation and chain elongation was differently affected at several steps. The morphological, biochemical and transcriptional changes induced by T3 in the tadpoles reared at 26 °C were almost completely abolished by exposure to cold temperature. Cold-induced arrest of amphibian metamorphosis may be due to the block of T3-signaling pathway at around the activation of primary TH-response genes. In nature, the bullfrog tadpoles overwinter with an increase in body mass during the coldest months of the year at high latitudes. This life stages may have a significant benefit in survival and fecundity.

## Methods

### Animals and experimental design

Tadpoles of the American bullfrog *L. catesbeianus* were collected from Saitama or Ibaraki, Japan, and commercially obtained. They were maintained in aerated and dechlorinated tap water at 22‒26 °C and were ad libitum fed boiled spinach three times a week. Their developmental stages were estimated according to the criteria of Taylor and Kollros [45]. After they were acclimated to laboratory conditions for 1‒2 weeks, tadpoles at stages X‒XIII were divided into four groups (6‒8 individuals/group) and placed in 6 L-aquaria with 4‒5.3 L of dechlorinated tap water. For the two cold-treated groups, two aquaria were moved into a cold room and the water temperature was gradually reduced to 4 °C at a rate of less than 1 °C/h, on the evening before starting experiments. For the two other groups, the water temperature in the remaining two aquaria was maintained at 26 °C as a control. The next morning, the experiments were started by adding T3 (final concentration 5 nM) into one aquarium at 4 and 26 °C and dimethyl sulfoxide (final 0.00025 %) into the other aquarium at 4 and 26 °C, and were continued for 3 or 7 days. Aquaria water was changed every day. Tadpoles were not fed during the experiment. Tadpoles were anesthetized by immersion in 0.2 % 3-aminobenzoic acid ethyl ester (Sigma, St. Louis, MO, USA), after which their body weight, body length, and tail length and height were measured, their blood was collected from the heart using heparinized capillaries, and their liver was dissected. The plasma was separated from the blood cells by centrifugation at 3000×*g* for 15 min at 4 °C and was stored at −35 °C for later use.

All housing and experimental procedures were conducted in accordance with the code of ethics of the Animal Welfare Committee of Shizuoka University.

### Measurements of plasma parameters

The concentration of glucose in plasma was determined by the mutarotase-glucose oxidase method [46] using a kit (Glucose C II-testWako), TGs by the glycerol-3-phosphate oxidase method [47] using a kit (Triglyceride E-testWako), cholesterol by the cholesterol oxidase method [48] using a kit (Cholesterol E-testWako), and free FAs by the acyl-CoA synthetase and acyl-CoA oxidase method [49] using a kit (NEFA C-testWako). All kits were used according to the manufacturer’s directions. Plasma parameters were quantified by scanning absorbance at 490 nm for glucose with a microplate reader (Emax, Molecular Devices, CA, USA), and absorbance at 600 nm for TGs and cholesterol and at 550 nm for free FAs with a spectrophotometer (U-3210, Hitachi Koki, Tokyo, Japan).

### Membrane lipid analysis

Lipids were extracted from tadpole liver according to the method described elsewhere [50]. In brief, liver from two tadpoles were pooled (0.2–0.4 g), added to methanol:chloroform (2:1, v/v; 0.3 mL) solvent, and crushed by a glass rod. After leaving the mixture at room temperature for 30 min, the supernatant was recovered by centrifugation at 1400×*g* for 10 min at 4 °C. The extract was washed with 0.9 % NaCl (1 mL) by agitating with a vortex mixer and centrifuging at 1400×*g* for 10 min at 4 °C. This process was repeated twice. The lower organic layer containing the extracted lipids was stored at −30 °C for analysis.

The extracted lipids (50 µL) were separated by TLC on a silica gel plate (KN3315756, Merck Millipore, Darmstadt, Germany) using a solvent system of chloroform:methanol:petroleum ether:acetone:acetic acid:water (20:15:10:5:1.3:1, v/v). The lipid spots were visualized under ultraviolet light after spraying with primuline (0.01 % in 80 % acetone). Each lipid spot was identified by co-chromatography with the standards: PC, PE, PS, PI, PG and CL. Each lipid spot was scraped off the silica gel plate and the silica powder was transferred to a screw-capped glass test tube. Pentadecanoic acid (100 μL of 50 μg/mL) was added to each tube as an internal standard, then methanolysis was carried out with 1 N methanolic HCl (1 mL; Sigma) for 30 min at 80 °C. Next, hexane (1 mL) and 0.9 % NaCl (1 mL) was added to the acidic solution and the solution was centrifuged at 1400×*g* for 10 min at 4 °C, resulting in the formation of two phases. The hexane layer was collected and evaporated by an evaporator (TC-8, TAITEC, Saitama, Japan). The residual FA methyl esters were re-suspended with hexane to 30 μL and analyzed by GC (QP2010SE, Shimadzu, Kyoto, Japan) equipped with a flame ionization detector on a capillary column (BPX5, 30 m × 0.25 mm; SGE Analytical Science, Melbourne, Australia). The column temperature was programmed at 240 °C. The injector and detector temperatures were 200 and 240 °C, respectively. The flow rate of carrier gas (He) was 0.4 mL/min.

### Preparation of mitochondria-rich fractions and enzyme assays

Mitochondria-rich fractions were prepared from the liver as described previously [51, 52], with some modifications. In brief, livers from two tadpoles were pooled (0.2–0.4 g), homogenized immediately in ice cold buffer (1 mL; 0.25 M sucrose, 1 mM ethylenediaminetetraacetic acid, 3 mM Tris–HCl, pH 7.5, and 1 mM reduced glutathione) in a Potter–Elvehjem homogenizer, and centrifuged at 500×*g* for 10 min at 4 °C. The superficial lipid layer was removed, and the supernatant was centrifuged at 9000×*g* for 10 min at 4 °C twice. The pellet containing mitochondria was re-suspended in homogenization buffer (0.3 mL) and stored at −80 °C until used.

COX activity was measured by monitoring the oxidation of reduced cytochrome c at 550 nm using a kit (Cytochrome C Oxidase Activity Assay Kit, BioChain, Newark, CA, USA), according to the manufacturer’s directions. All assays were started by mixing the mitochondria-rich fraction (50 μL; 12.5 μg protein), enzyme assay buffer (425 μL), and reduced cytochrome c (25 μL), and were done at 25 °C in triplicate using a spectrophotometer. Activity was calculated by the decrease in absorbance at 550 nm using an extinction coefficient of 21.84 mmol/L/cm (manufacturer’s protocol) between ferrocytochrome c and ferricytochrome c and is expressed as μmoles/min (U) cytochrome c transformed/mg protein or U/g liver (first order reaction).

CPT activity was measured according to the method described elsewhere [51, 53]. Enzyme assay solution [250 μL of 116 mM Tris–HCl, pH 8.0, 2.5 mM ethylenediaminetetraacetic acid, 0.2 % Triton X-100, 0.5 mM 5,5′-dithiobis (2-nitrobenzoic acid)] was mixed with the mitochondria-rich fraction (20 μL; 20 μg protein) and distilled water (210 μL) in a cuvette. Next, 2 mM palmitoyl-CoA (10 μL) and 62.5 mM l-carnitine (10 μL; or enzyme assay solution for blank assay) were added to the mixture. Absorbance at 412 nm was monitored immediately at 0.2 min intervals with a spectrophotometer and the production of 5-thio-2-nitrobenzoic acid was calculated using an extinction coefficient of 13.6 mmol/L/cm [51]. CPT activity was determined as an l-carnitine-dependent rate that was obtained from the difference between the rates with and without l-carnitine, and is expressed as mU produced 5-thio-2-nitrobenzoic acid/mg protein or mU/g liver.

The protein content of the mitochondria-rich fractions was estimated by the micro-Lowry method [54] with bovine serum albumin as the standard using a microplate reader.

### RNA extraction and RT-qPCR analysis

Tadpole liver (~0.1 g) was lysed with guanidinium thiocyanate solution (1000 μL) [55]. The total RNA was isolated with phenol and chloroform. To confirm its integrity, RNA (1 μg per lane) was electrophoresed in a 1 % agarose gel containing 2.0 M formaldehyde, and 28S and 18S rRNAs were visualized by ethidium bromide staining. Complementary DNAs were synthesized from the total RNA (200 ng) in 10 μL of 1× Taqman RT buffer using Taqman RT reagents kit (Applied Biosystems, Foster City, CA, USA) according to the manufacturer’s instructions. The expression of individual genes was estimated in triplicate using Power SYBR Green Master Mix and ABI Prism 7000 sequence detection System (Applied Biosystems) with a specific primer set (each 200 nM), as shown previously [5]. Detailed information of primer sets and RT-qPCR conditions is shown in Additional files 5 and 6: Tables S3 (Additional file 5) and S4 (Additional file 6). We included controls lacking cDNA template to determine the specificity of target cDNA amplification. All assays produced unique dissociation curves. The endpoint used in real-time PCR quantification, C_q_, was defined as the PCR cycle number that crosses an arbitrarily placed signal threshold and is a function of the amount of target DNA present in the starting material. Quantification was determined by applying the 2^−Cq^ formula and calculating the average of the values obtained for each sample. Eligibility of this formula was verified by qPCR using RT-qPCR or RT product of total RNA as a template at different concentrations that covered 3–5 orders of magnitude. To standardize each experiment, the amounts of the test transcripts were divided by those of reference genes in the same sample by the 2^−ΔΔCq^ method [56]. As the Cq values for the β-actin (actb) transcript was more invariable among the four experimental groups than those for the ribosomal protein L8 (rpl8) and lactate dehydrogenase B (ldhb) transcripts, we used the actb as a reference gene.

### Statistical analysis

All assay data are presented as mean ± standard error of the mean (SEM). Differences between groups were analyzed by one-way or two-way analysis of variance using the Fisher’s test for multiple comparisons to show significant differences. *p* < 0.05 was considered statistically significant.

## Additional files

10.1186/s13578-016-0087-5 Morphological changes of the tadpoles exposed to cold and 3,3′,5-triiodothyronine (T3).
10.1186/s13578-016-0087-5 Changes in compositions of fatty acids in phosphatidylcholine, phosphatidylethanolamine, phosphatidylserine, phosphatidylinositol, phosphatidylglycerol and cardiolipin from the tadpole liver.
10.1186/s13578-016-0087-5 Pathways of glycerophospholipid synthesis (*A*) and very long chain fatty acid desaturation and elongation (*B*) related to the transcription data. Transcription data and full names of genes are shown in Fig. 7 and its legend. Transcription regulatory protein is boxed in green, hepatic lipids and fatty acids analyzed (see Table 1 and Fig. 2) are boxed. 3,3′,5-Triiodothyronine-response and cold-response genes on day 3 or day 7 are marked in red and blue, respectively. PC, phosphatidylcholine; PE, phosphatidylethanolamine; PS, phosphatidylserine; PI, phosphatidylinositol; PG, phosphatidylglycerol; CL, cardiolipin; Cho-p, choline phosphate; CDP-Cho, cytidine diphosphate choline; CDP-Etn, cytidine diphosphate ethanolamine. PA, palmitic acid; POA, palmitoleic acid; SA, stearic acid; OA, oleic acid; LA, linoleic acid; GLA, γ-linolenic acid; AA, arachidonic acid; ALA, α-linolenic acid; EPA, eicosapentaenoic acid; DPA, docosapentaenoic acid; DHA, docosahexaenoic acid.
10.1186/s13578-016-0087-5 Energy metabolic pathways related to the transcriptional data. Transcription data and full names of genes are shown in Fig. 6 and its legend. Transcription regulatory proteins are boxed in green, plasma biochemical components analyzed (see Fig. 1) are boxed. Enzyme activities examined are underlined. 3,3′,5-Triiodothyronine-response and cold-response genes on day 3 or day 7 are marked in red and blue, respectively.
10.1186/s13578-016-0087-5 The list of primers used in quantitative polymerase chain reaction.
10.1186/s13578-016-0087-5 Detailed quantitative polymerase chain reaction information.

## Abbreviations

CL: cardiolipin
COX: cytochrome c oxidase
CPT: carnitine palmitoyltransferase
FA: fatty acid
GC: gas chromatography
MUFA: monounsaturated fatty acid
PC: phosphatidylcholine
PE: phosphatidylethanolamine
PG: phosphatidylglycerol
PI: phosphatidylinositol
PS: phosphatidylserine
PUFA: polyunsaturated fatty acid
RT-qPCR: reverse transcription-quantitative polymerase chain reaction
SEM: standard error of the mean
SFA: saturated fatty acid
T3: 3,3′,5-triiodothyronine
TG: triglyceride
TH: thyroid hormone
TLC: thin-layer chromatography
TR: thyroid hormone receptor
UI: unsaturation index
