# Supplementary material for: Cold temperature blocks thyroid hormone-induced changes in lipid and energy metabolism in the liver of Lithobates catesbeianus tadpoles
Source: Cell Biosci. 2016 Mar 15;6:19. doi: 10.1186/s13578-016-0087-5 (PMC4792105; doi:10.1186/s13578-016-0087-5)
Supplement: Supplementary file 3 — 10.1186/s13578-016-0087-5 Pathways of glycerophospholipid synthesis (A) and very long chain fatty acid desaturation and elongation (B) related to the transcription data. Transcription data and full names of genes are shown in Fig. 7 and its legend. Transcription regulatory protein is boxed in green, hepatic lipids and fatty acids analyzed (see Table 1 and Fig. 2) are boxed. 3,3′,5-Triiodothyronine-response and cold-response genes on day 3 or day 7 are marked in red and blue, respectively. PC, phosphatidylcholine; PE, phosphatidylethanolamine; PS, phosphatidylserine; PI, phosphatidylinositol; PG, phosphatidylglycerol; CL, cardiolipin; Cho-p, choline phosphate; CDP-Cho, cytidine diphosphate choline; CDP-Etn, cytidine diphosphate ethanolamine. PA, palmitic acid; POA, palmitoleic acid; SA, stearic acid; OA, oleic acid; LA, linoleic acid; GLA, γ-linolenic acid; AA, arachidonic acid; ALA, α-linolenic acid; EPA, eicosapentaenoic acid; DPA, docosapentaenoic acid; DHA, docosahexaenoic acid. [file 13578_2016_87_MOESM3_ESM.pdf]

Fig. S1

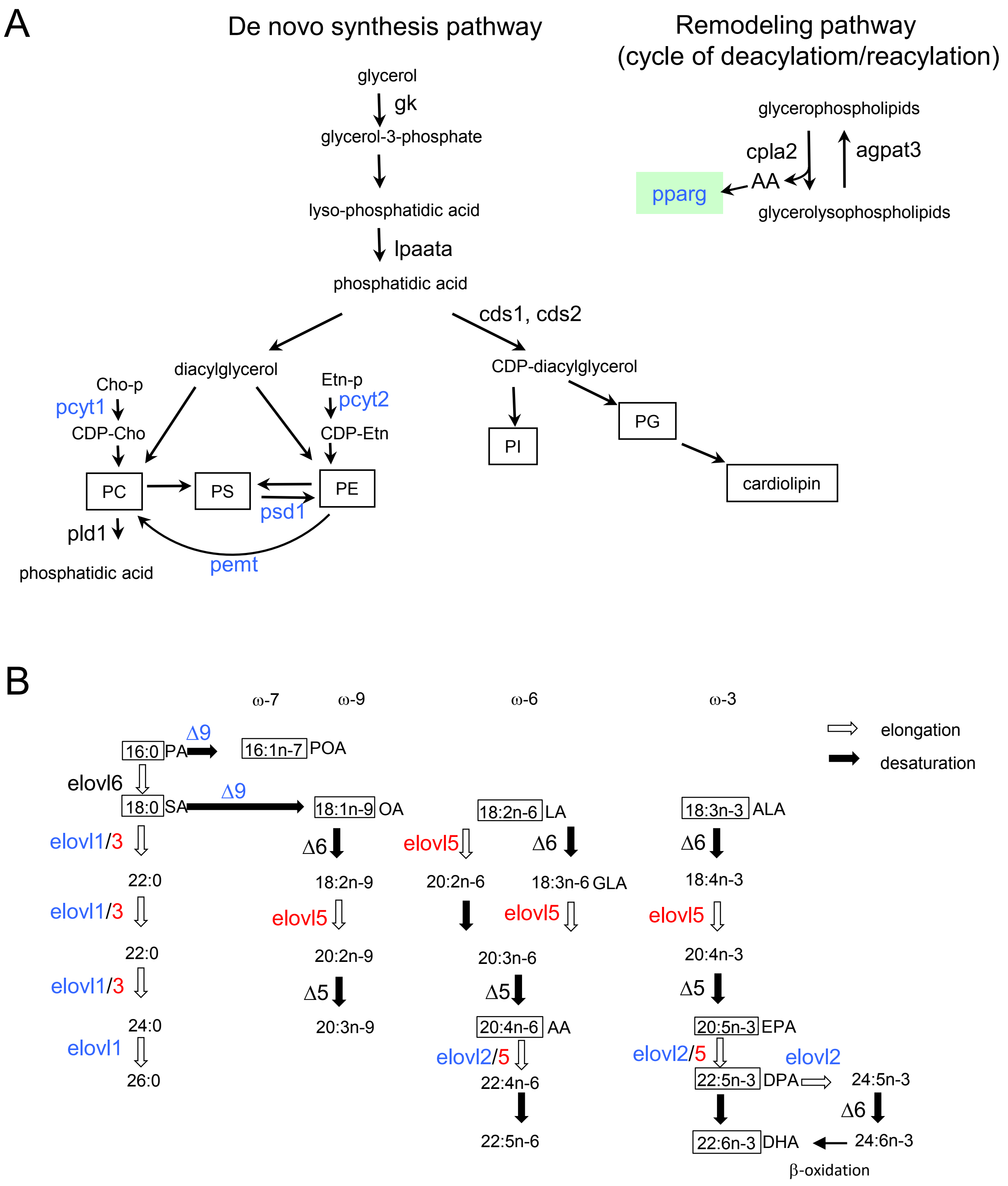

Fig. S1. Pathways of glycerophospholipid synthesis (*A*) and very long chain fatty acid desaturation and elongation (*B*) related to the transcription data. Transcription data and full names of genes are shown in Fig. 7 and its legend. Transcription regulatory protein is boxed in green, hepatic lipids and fatty acids analyzed (see Table 1 and Fig. 2) are boxed. 3,3',5-Triiodothyronine-response and cold-response genes on Day 3 or Day 7 are marked in red and blue, respectively. PC, phosphatidylcholine; PE, phosphatidylethanolamine; PS, phosphatidylserine; PI, phosphatidylinositol; PG, phosphatidylglycerol; CL, cardiolipin; Cho-p, choline phosphate; CDP-Cho, cytidine diphosphate choline; CDP-Etn, cytidine diphosphate ethanolamine. PA, palmitic acid; POA, palmitoleic acid; SA, stearic acid; OA, oleic acid; LA, linoleic acid; GLA, γ-linolenic acid; AA, arachidonic acid; ALA, α-linolenic acid; EPA, eicosapentaenoic acid; DPA, docosapentaenoic acid; DHA, docosahexaenoic acid.
