# Supplementary material for: Cold temperature blocks thyroid hormone-induced changes in lipid and energy metabolism in the liver of Lithobates catesbeianus tadpoles
Source: Cell Biosci. 2016 Mar 15;6:19. doi: 10.1186/s13578-016-0087-5 (PMC4792105; doi:10.1186/s13578-016-0087-5)
Supplement: Supplementary file 4 — 10.1186/s13578-016-0087-5 Energy metabolic pathways related to the transcriptional data. Transcription data and full names of genes are shown in Fig. 6 and its legend. Transcription regulatory proteins are boxed in green, plasma biochemical components analyzed (see Fig. 1) are boxed. Enzyme activities examined are underlined. 3,3′,5-Triiodothyronine-response and cold-response genes on day 3 or day 7 are marked in red and blue, respectively. [file 13578_2016_87_MOESM4_ESM.pdf]

Fig. S2

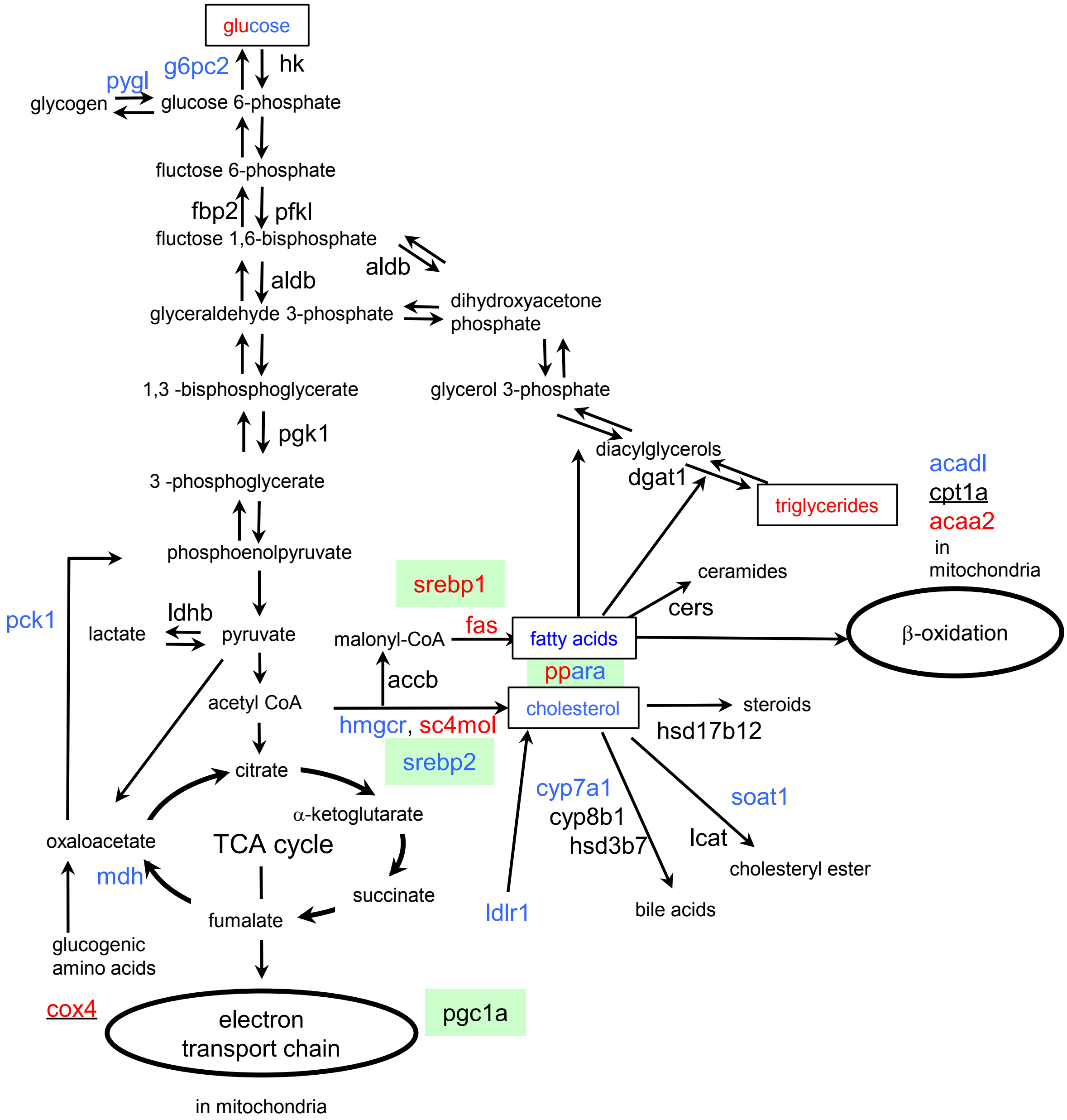

Fig. S2. Energy metabolic pathways related to the transcriptional data. Transcription data and full names of genes are shown in Fig. 6 and its legend. Transcription regulatory proteins are boxed in green, plasma biochemical components analyzed (see Fig. 1) are boxed. Enzyme activities examined are underlined. 3,3',5-Triiodothyronine-response and cold-response genes on Day 3 or Day 7 are marked in red and blue, respectively.
