# Supplementary material for: Cold temperature blocks thyroid hormone-induced changes in lipid and energy metabolism in the liver of Lithobates catesbeianus tadpoles
Source: Cell Biosci. 2016 Mar 15;6:19. doi: 10.1186/s13578-016-0087-5 (PMC4792105; doi:10.1186/s13578-016-0087-5)
Supplement: Supplementary file 6 — 10.1186/s13578-016-0087-5 Detailed quantitative polymerase chain reaction information. [file 13578_2016_87_MOESM6_ESM.docx]

Table S4. Detailed quantitative polymerase chain reaction information.

**_________________________________________________________________________________________________________________________________________________________________**

**Experimenal design**

Definition of experimental and control groups 26°C (-)T3 group: The rearing water without T3 in the aquaria was maintained at 26°C.

26°C (+)T3 group: The rearing water with 5 nM T3 in the aquaria was maintained at 26°C.

4°C (-)T3 group: The rearing water without T3 in the aquaria was maintained at 4°C.

4°C (+)T3 group: The rearing water with 5 nM T3 in the aquaria was maintained at 4°C.

Number within each group n = 6-8

**Sample**

Description The liver of *Lithobates catesbeianus* tadpoles collected from Saitama or Ibaraki, Japan

**Nucleic acid extraction**

Procedure and/or instrumentation RNA extraction: the AGPC method (Chomczynski and sacchi, 1987)

Details of DNase or RNase treatment After adding 1 U/μL RQ1 DNase, incubated for 30 min at 37 °C (Roche, Tokyo, Japan).

Contamination assessment (DNA or RNA) The Cq values of DNase-treated samples were slightly higher than those of untreated samples.

Nucleic acid quantification Concentrations, 200-500 μg/mL; volume, 100 μL

Instrument and method Instrument, BioSpec-nano (Shimadzu, Kyoto, Japan); Method, Instruction manual

Purity (A260/A280) A_260_/A_280_: 1.90-2.10

RNA integrity: method/instrument 28S rRNA and 18S rRNA bands were detected on agarose gel electrophoresis

Method Electrophoresis in a 1% agarose gel containing 2 M formaldehyde

**Reverse transcription**

Complete reaction conditions Final concentration of reaction reagents:

20 ng/μL RNA sample,

1 xTaqman RTbuffer,

5.5 mM MgCl_2_,

500 μM each dNTP

2.5 μM Random hexamers,

0.4 U/μL RNase inhibitor,

1.25 U/μL MultiScribe reverse transcriptase

Amount of RNA and reaction volume Amount of RNA, 200 ng; reaction volume, 10 μL

Priming oligonucleotide (if using GSP) and concentration Random hexamers; final concentration, 2.5 μM

Reverse transcriptase and concentration MultiScribe reverse transcriptase; final concentration, 1.25 U/μL

Temperature and time 25°C for 10 min, 48°C for 30 min, and then 95°C for 5 min

Manufacturer of reagents and catalogue numbers Taqman RT reagent kit

Applied Biosystems (Foster City, CA, USA), Cat. No.,N8080234

Storage conditions of cDNA -20°C

**qPCR target information**

Gene symbol See the text and Table S3

Sequence accession number See Table S3

Location of amplicon See Table S3

Amplicon length See Table S3

In silico specificity screen (BLAST, and so on) The primer specificity was confirmed using BLAST search.

**PCR oligonucleotides**

Primer sequences See Table S3

**qPCR protocol**

Complete reaction conditions Final volume of reaction reagents:

12.5 μL 2 x Power SYBR Green Master Mix,

2 μL 2.5 μM each Primer,

8.5 μL Diethylpyrocarbonate-treated water,

2 μL cDNA mixture

Reaction volume and amount of cDNA/DNA Reaction volume, 25 μL; cDNA mixture after reverse transcription, 2 μL

Primer, (probe), Mg^2+^, and dNTP concentrations Primers, each 200 nM (final conc.)

Power SYBR Green Master Mix includes Mg^2+^ and dNTP, whose concentrations are unclear.

Polymerase identity and concentration Power SYBR Green Master Mix includes AmpliTaq Gold DNA Polymerase, whose concentration is unclear.

Buffer/kit identity and manufacturer Power SYBR Green Master Mix,

Applied Biosystems (Foster City, CA, USA), Cat. No. 4367659

Exact chemical composition of the buffer Unclear

Additives (SYBR Green I, DMSO, and so forth) Power SYBR Green Master Mix includes SYBR Green I and Passive reference, whose concentrations are unclear.

Manufacturer of plates/tubes and catalog number ABgene PCR Detection plates (Thermo Scientific, Yokohama, Japan), Cat. No. AB-1100

Complete thermocycling parameters 95°C for 10 min, and 40 cycles of 95°C for 15 s, 60°C for 1 min, and 50°C for 2 min

Manufacturer of qPCR instrument ABI Prism 7000 Sequence Detection System (Applied Biosystems, Foster City, CA, USA)

**qPCR validation**

Specificity (gel, sequence, melt, or digest) Single band on agarose gel with an expected size, and single peak of melting curve

PCR efficiency calculated from slope See Table S3

r^2^ of calibration curve See Table S3

Linear dynamic range 1:10:100:1000:10000:100000

**Data analysis**

qPCR analysis program (source, version) ABI PRISM 7000 SDS software version 1.0 (build 81 rev3)

Method of Cq determination Following the instruction manual, Cqs were arbitrarily determined the appropriate position.

Outlier identification and disposition None

Results for NTCs See Table S3

Description of normalization method The 2^-ΔΔCq^ method (Livak and Schmittgen, 2001)

Number and stage (reverse transcription orqPCR) of technical replicates 3 times every steps (RNA extraction, reverse transcription and qPCR)

Repeatability (intraassay variation) In triplicates

Statistical methods for results significance Fisher's test

Software (source, version) Microsoft Excel 2003 Data Analysis

**__________________________________________________________________________________________________________________________________________________**
